# Updated Recommendations for Use of Tetanus Toxoid, Reduced Diphtheria Toxoid, and Acellular Pertussis Vaccine (Tdap) in Pregnant Women — Advisory Committee on Immunization Practices (ACIP), 2012

**Published:** 2013-02-22

**Authors:** Mark Sawyer, Jennifer L. Liang, Nancy Messonnier, Thomas A. Clark

**Affiliations:** Univ of California, San Diego, La Jolla, California; Div of Bacterial Diseases, National Center for Immunization and Respiratory Diseases, CDC

In October 2011, in an effort to reduce the burden of pertussis in infants, the Advisory Committee on Immunization Practices (ACIP) recommended that unvaccinated pregnant women receive a dose of tetanus toxoid, reduced diphtheria toxoid, and acellular pertussis vaccine (Tdap) (1). Vaccination of women with Tdap during pregnancy is expected to provide some protection to infants from pertussis until they are old enough to be vaccinated themselves. Tdap given to pregnant women will stimulate the development of maternal antipertussis antibodies, which will pass through the placenta, likely providing the newborn with protection against pertussis in early life, and will protect the mother from pertussis around the time of delivery, making her less likely to become infected and transmit pertussis to her infant (1). The 2011 Tdap recommendation did not call for vaccinating pregnant women previously vaccinated with Tdap. On October 24, 2012, ACIP voted to recommend use of Tdap during every pregnancy. This report summarizes data considered and conclusions made by ACIP and provides guidance for implementing its recommendations. These updated recommendations on use of Tdap in pregnant women aim to optimize strategies for preventing pertussis morbidity and mortality in infants.

ACIP is chartered as a federal advisory committee to provide expert external advice and guidance to the Director of the Centers for Disease Control and Prevention (CDC) on use of vaccines and related agents for the control of vaccine-preventable diseases in the civilian population of the United States. Recommendations for routine use of vaccines in children and adolescents are harmonized to the greatest extent possible with recommendations made by the American Academy of Pediatrics, the American Academy of Family Physicians (AAFP), and the American College of Obstetricians and Gynecologists. Recommendations for routine use of vaccines in adults are reviewed and approved by the American College of Physicians, AAFP, the American College of Obstetricians and Gynecologists, and the American College of Nurse-Midwives. ACIP recommendations adopted by the CDC Director become agency guidelines on the date published in the *Morbidity and Mortality Weekly Report* (*MMWR*).

The United States has experienced substantial increases in reported pertussis cases over the past several years. Provisional case counts for 2012 have surpassed the last peak year, 2010, with 41,880 pertussis cases and 14 deaths in infants aged <12 months (2) (CDC, unpublished data, 2012). To reduce this burden, optimizing the current vaccination program and protecting infants who are at highest risk for death are immediate priorities. Since the 2011 ACIP vaccination recommendation, uptake of Tdap among pregnant women has been low; one survey of 1,231 women (August 2011 to April 2012) estimated that only 2.6% of women received Tdap during their recent pregnancy (3). New data indicate that maternal antipertussis antibodies are short-lived; therefore, Tdap vaccination in one pregnancy will not provide high levels of antibodies to protect newborns during subsequent pregnancies (4).

## Methods

In monthly teleconferences during 2012, the ACIP Pertussis Vaccines Work Group considered published, peer-reviewed literature and unpublished data relevant to vaccinating pregnant women with Tdap. When data were not available, expert opinion was considered. Summaries of the data reviewed and work group discussions were presented to ACIP before recommendations were proposed. The proposed Tdap recommendation for pregnant women was presented at the October 2012 ACIP meeting and approved by ACIP.

## Summary of ACIP Deliberations and Rationale

### A dose of Tdap during each pregnancy

Very young infants are dependent solely on maternal antibodies and lack the ability to mount a cell-mediated response (4). The effectiveness and optimal concentration of maternal antipertussis antibodies in newborns are not yet known, but high levels of antibodies in the first weeks after birth likely confer protection and might prevent pertussis or modify disease severity (5–7). Studies on the persistence of antipertussis antibodies following a dose of Tdap show antibody levels in healthy, nonpregnant adults peak during the first month after vaccination, with substantial antibody decay after 1 year (8–10). Antibody kinetics in pregnant women likely would be similar. One study evaluated persistence of maternal antipertussis antibody concentrations from maternal delivery and cord blood pairs from women who received Tdap within the prior 2 years (4). The estimated antipertussis antibody concentrations at birth in most of these infants were considered unlikely to provide adequate protection. These findings indicate that maternal antibodies from women immunized before pregnancy waned quickly and the concentration of maternal antibodies was unlikely to be high enough to provide passive protection to infants (4). Because antibody levels wane substantially during the first year after vaccination, ACIP concluded a single dose of Tdap at one pregnancy would be insufficient to provide protection for subsequent pregnancies.

### Potential Impact of Tdap During Pregnancy

For the 2011 ACIP recommendation, ACIP reviewed a decision analysis model developed to assess the impact and cost effectiveness of Tdap vaccination during pregnancy compared with immediately postpartum vaccination (1). The model showed that Tdap vaccination during pregnancy would prevent more infant cases, hospitalizations, and deaths compared with the postpartum dose (11).

For this updated recommendation, the model was rereviewed and the analysis updated. To estimate the potential impact of Tdap given either during pregnancy or postpartum, percent mean reductions were applied to the annual mean number of reported pertussis cases in infants aged <12 months during 2000–2011 (CDC, unpublished data, 2011). During 2000–2011, the annual mean of pertussis cases in infants aged <12 months was 2,746 (range: 1,803–4,298), hospitalizations was 1,217 (range: 687–1,938), and deaths was 18 (range: 8–35) (CDC, unpublished data, 2011). Based on the model, Tdap vaccination during pregnancy might prevent 906 (range: 595–1,418) infant cases, 462 (range: 261–736) hospitalizations, and nine (range: 4–17) deaths; a postpartum dose might prevent 549 (range: 361–860) infant cases, 219 (range: 124–349) hospitalizations, and three (range: 1–6) deaths (CDC, unpublished data, 2012).

### Birth Statistics in the United States

To address the likelihood that women might receive Tdap during consecutive pregnancies in a short period, and therefore theoretically be at greater risk for adverse reactions, ACIP reviewed available data on birth statistics. In the United States, approximately 4 million births are reported each year, and an average of 2.06 children are born per woman in a lifetime (12,13). Among women with more than one pregnancy, only 2.5% have an interval ≤12 months between births (14). The majority of women, who have two pregnancies, have an interval of ≥13 months between births (14). For women of lower socioeconomic status, the interval between pregnancies generally is ≥18 months (15). Approximately 5% of women have four or more babies (16). ACIP concluded that the interval between subsequent pregnancies is likely longer than the persistence of maternal antipertussis antibodies, and were reassured that most women would receive only 2 Tdap doses and a small proportion of women would receive ≥4 doses of Tdap.

### Safety of Repeat Tdap Administration to Pregnant Women

In 2011, ACIP concluded that available data did not suggest any elevated frequency or unusual patterns of adverse events in pregnant women who received Tdap and that the few serious adverse events reported were unlikely to have been caused by the vaccine; at that time, a dose of Tdap for every pregnancy was not considered (9). Published data on receipt of 2 doses of Tdap and multiple doses of tetanus toxoid–containing vaccines were reviewed. Receipt of a second dose of Tdap at a 5- or 10-year interval in healthy nonpregnant adolescents and adults was well tolerated; injection site pain was the most commonly reported adverse event (9,17–20). The frequency of reported adverse events for the second dose was similar to the first dose in these same subjects and in naïve controls receiving Tdap for the first time. Of the few serious adverse events reported, none were attributed to the vaccine. Fever was reported in 2.4%–6.5% of recipients of a Tdap booster; the frequency of fever was similar to that in the same subjects after their first Tdap dose and in naïve controls (9,17–19). Studies on short intervals (i.e., within 21 days or ≤2 years) between receipt of tetanus and diphtheria toxoids (Td) and Tdap or Tdap-inactivated polio vaccine in healthy, nonpregnant adolescents and adults found no serious adverse events (21–23). Fever was reported in 1.7%–6.8% of subjects who received Tdap ≤2 years after Td; rates were comparable to the control group and to cohorts that received Tdap longer after receipt of Td (21,22). The number of subjects in these studies was small, and therefore, the findings do not rule out the possibility of rare but serious adverse events.

A theoretical risk exists for severe local reactions (e.g., Arthus reactions, whole limb swelling) for pregnant women who have multiple closely spaced pregnancies. Arthus reactions and whole limb swelling are hypersensitivity reactions that have been associated with vaccines containing tetanus toxoid, tetanus and diphtheria toxoids, and/or pertussis antigens. Historical data on multiple doses of Td and tetanus toxoid vaccines (TT) indicate that hypersensitivity was associated with higher levels of preexisting antibody (24–26). The frequency of side effects depended on antigen content, product formulation, preexisting antibody levels related to the interval since last dose, and the number of doses (24–26). Challenges to reviewing historical data on multiple doses of TT and Td include differences in adjuvant and toxoid amounts in vaccines over time and severity of adverse events by number of vaccines received (24–26). Most of the data are historical, and the risk for severe adverse events likely has been reduced with current formulations that contain lower doses of TT.

TT and Td have been used extensively in pregnant women worldwide to prevent neonatal tetanus; large studies on use of TT during pregnancy have not reported clinically significant severe adverse events (27–30). Safety data on use of Td during multiple pregnancies have not been published. ACIP believes the potential benefit of preventing pertussis morbidity and mortality in infants outweighs the theoretical concerns of possible severe adverse events.

ACIP concluded that experience with tetanus-toxoid containing vaccines suggests no excess risk for severe adverse events for women receiving Tdap with every pregnancy. ACIP stated the need for safety studies of severe adverse events when Tdap is given during subsequent pregnancies. Plans for safety monitoring in pregnant women following Tdap administration include enhanced monitoring in Vaccine Adverse Event Reporting System (VAERS) and utilizing the Vaccine Safety Datalink (VSD) to assess acute adverse events, adverse pregnancy outcomes affecting the mother, and birth outcomes; assessing risks for rare adverse events in pregnant women after Tdap will require data collection for several years (31).

### Vaccination During the Third Trimester

Tdap may be administered any time during pregnancy, but vaccination during the third trimester would provide the highest concentration of maternal antibodies to be transferred closer to birth (4). After receipt of Tdap, a minimum of 2 weeks is required to mount a maximal immune response to the vaccine antigens (32,33). Active transport of maternal immunoglobulin G does not substantially take place before 30 weeks of gestation (34). One study of pregnant women who received Tdap within the prior 2 years noted that maternal antibodies waned quickly; even women immunized during the first or second trimester had low levels of antibodies at term (4). Therefore, to optimize the concentration of vaccine-specific antipertussis antibodies transported from mother to infant, ACIP concluded that pregnant women should be vaccinated with Tdap during the third trimester.

## ACIP Recommendations for Pregnant Women

ACIP recommends that providers of prenatal care implement a Tdap immunization program for all pregnant women. Health-care personnel should administer a dose of Tdap during each pregnancy, irrespective of the patient’s prior history of receiving Tdap.

### Guidance for Use

To maximize the maternal antibody response and passive antibody transfer to the infant, optimal timing for Tdap administration is between 27 and 36 weeks gestation although Tdap may be given at any time during pregnancy. For women not previously vaccinated with Tdap, if Tdap is not administered during pregnancy, Tdap should be administered immediately postpartum.

### Special Situations

#### Pregnant women due for tetanus booster

If a tetanus and diphtheria booster vaccination is indicated during pregnancy (i.e., >10 years since previous Td), then Tdap should be administered. Optimal timing is between 27 and 36 weeks gestation to maximize the maternal antibody response and passive antibody transfer to the infant.

#### Wound management for pregnant women

As part of standard wound management to prevent tetanus, a tetanus toxoid–containing vaccine might be recommended for wound management in a pregnant woman if ≥5 years have elapsed since the previous Td booster. If a Td booster is recommended for a pregnant woman, health-care providers should administer Tdap.

#### Pregnant women with unknown or incomplete tetanus vaccination

To ensure protection against maternal and neonatal tetanus, pregnant women who never have been vaccinated against tetanus should receive three vaccinations containing tetanus and reduced diphtheria toxoids. The recommended schedule is 0, 4 weeks, and 6 through 12 months. Tdap should replace 1 dose of Td, preferably between 27 and 36 weeks gestation to maximize the maternal antibody response and passive antibody transfer to the infant.

### Cocooning

ACIP recommends that adolescents and adults (e.g., parents, siblings, grandparents, child-care providers, and health-care personnel) who have or anticipate having close contact with an infant aged <12 months should receive a single dose of Tdap to protect against pertussis if they have not received Tdap previously. Guidance will be forthcoming on revaccination of persons who anticipate close contact with an infant, including postpartum women who previously have received Tdap.

## Research Needs

Future research needs will address the effectiveness of Tdap vaccination of pregnant women to prevent infant pertussis morbidity and mortality, the impact of timing of Tdap during pregnancy on infant pertussis, and safety of multiple doses of Tdap in pregnant women. CDC will monitor and assess the safety of Tdap use during pregnancy. Results from these studies and monitoring systems will inform future considerations made by ACIP on use of Tdap in preventing infant pertussis morbidity and mortality.
